# The Different Models of Community Eldercare Service in China

**DOI:** 10.3389/fsoc.2019.00007

**Published:** 2019-02-14

**Authors:** Lei Zhang, Jinwei Yang

**Affiliations:** ^1^The School of Law, Chengdu University, Chengdu, China; ^2^Research Institute of Social Development, Southwestern University of Finance and Economics, Chengdu, China

**Keywords:** community eldercare service, older people, comparative analysis, case study, China

## Abstract

The study examines the development trajectory of community eldercare service and how the local governments pursue the policy practice in delivering community eldercare in China. After reviewing the pilot practice in different localities, this paper will attempt to compare and classify the models of community eldercare service and find out the different features and outcomes of the models. Through the comparative study of community eldercare model, this paper will provide the policy implications for implementing national community eldercare system in the future.

## Introduction

**Figure 1:**
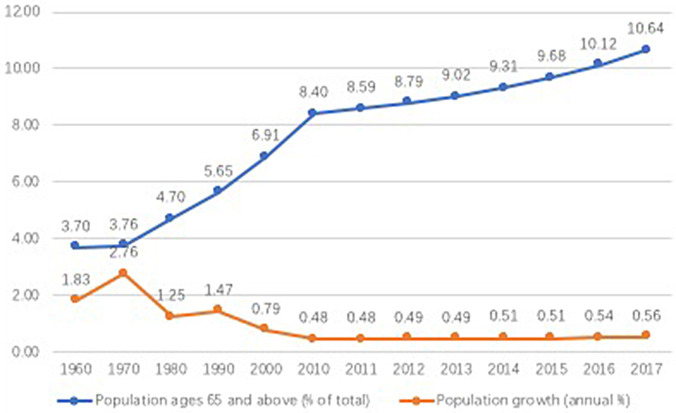
The demographic aging in China (1960–2017).

As the population ages, “older people care” becomes an inevitable global problem. According to the latest China statistic of 2017, the number of aged 60 and above was 240.9 million, accounting for 17.3% of the whole population. The number of population aged 65 and above was 158.31 million, taking up 11.4% of the total population [National Bureau of Statistics (NBS), 2017]. The speed of demographic aging has been increasing over time. The Figure 1 shows that population ages 65 and above account of 3.70% of total population in 1960 and started to rise gradually, particularly from 2010 the percentage of the population aged 65 and above has increased to 8.4%, and still increase over time. Whereas, the annual population growth started to decrease from 2.76% in 1979 to 1.25% at the 1980s because of the one-child policy. After coming into the twenty-first century, the annual growth rate keeps at a stable low level of around 5%. All these indicators show that China is marching toward deeper population aging. The challenge of eldercare is increasingly severe.

Family support as the traditional older people care could not be adapted to the fast-changing social, economic demands. The greater population mobility, decreasing dependency ratio, generation idea conflicts, as well as attitude change toward older people support to make the family support of, older people less possible in contemporary China. Private eldercare institution is the option of the wealthy aging population. This institutional eldercare is not accessible to the majority of older people because of the limitations such as high cost, entrance restriction, or the traditional value bias of “filial piety.” Therefore, both the family support of older people and the private institutional eldercare have disadvantages and problems and they could not be the feasible option for Chinese older people. Facing the challenges of rapid aging, government and society have tried different options to deal with this problem.

Community eldercare is a service mode in which older people living at home and the community provides day care in the form of visiting service or staying in a daycare center. Under this model, older people do not need to move out of the home and could get support from family members at times. Meanwhile, they could live in the familiar community and receive the care service from the community. This eldercare service is an innovation combining family support and social support to provide service for older people. Since the 1980s, there is an increasing amount of research on this eldercare model, and there is a consensus that this model could solve the aging problem of China in transition (Peng, 2010; Ding, 2013; Tong, 2015).

## Research Rational and Research Questions

There has been a recent shift in reforming the welfare system associated with the aging population; the focus now is not only on the financial sustainability of the system but also the solution for the senior population. Most developed countries try to meet these needs with a complex system of different service through privatization and decentralization. In this paper, we firstly review the development of the community eldercare service in China and then examine the community eldercare practice mode in the sub-national level: Chongqing, Qingdao, Guangzhou, and Beijing. The policy reviews demonstrate the development trajectory of community eldercare service and national reform direction in the past decades. The local practice cases illustrate the various policy innovations in the system design and implementation. Beyond that, the study also attempts to engage with the welfare service system discussion. The comparison and typology of the community eldercare system based on generosity and accessibility would facilitate the implementation of national community eldercare service in future.

## Research Method: Comparative Case Studies

This study adopts the comparative case study to interpret the implications of similarities and differences across the local cases selected in order to guide further implementation of the national system. Comparative case studies cover two or more cases in a way that produces more generalizable knowledge and compare within and across contexts. Comparative case studies involve the analysis of the similarities, differences, and patterns across two or more cases that share a common goal. An understanding of each case is the foundation for the comparative analysis.

The cases selected including Chongqing, Qingdao, Guangzhou, and Beijing cities. The reasons for case selection are as follows. First, the four cases represent different degrees of demographic aging: until 2016 population aged 60 and above takes up 24% of the total population in Beijing (GOV, 2017). The aging rate; n Chongqing and Qingdao is, respectively, 20.76 and 20.60% (GOV, 2016). Among the four cases, Guangzhou has the lowest aging rate of 18.03% [Guang Zhou Min Zheng (GZMZ), 2018]. Second, the four cases are geographically located in the east, middle, and west of China, which represents different economic development levels and openness of the economy. Third, the four cases represent the dominant community eldercare model emerging at the local level. They are the typical models with obvious and distinguishing features in delivering community eldercare service. The other cities practices are developed on the basis of a variety of these four models. Therefore, this study examines and the four cases in details and attempts to construct a typology of community eldercare based on comparative welfare framework, in a way to demonstrate the general picture of community eldercare service in China and as well as tell differences to give effective implication for future national implementation.

The qualitative and quantitative data are used in comparative case studies. In order to generate a good understanding of China's context as well as each local city, this study adopted the fieldwork visits, interviews, and document analysis to collect various data.

## The Theoretical Framework of Comparing the Community Eldercare Service

The comparative case studies require more extensive conceptual and analytic work. In order to join in the comparative welfare discussion, we have to synthesize the across cases beyond the comparison of similarities and differences in practice details. When facing a different variety of systems that are vertically as well as horizontally fragmented, policymakers and researchers need systematic information for policy learning. The typology of different systems is an effective way to compare and learn from the sophisticated institutional design.

The literature on the typology of welfare regime is not novel now, more recent studies shift to classify the specific welfare systems (e.g., the pension system, healthcare system, long-term care system) with the aim to collect more systematized information to do comparison or policy learning. The previous studies attempt to group the long-term care (LTC) system from a different perspective (Anttonen and Jorma, 1996; Wittenberg et al., 2002; Bettio and Janneke, 2004), there is no consensus on the framework and method to classify the LTC system. The information on national divergence in LTC system is harder to obtain than the pension or health care system. Also, the lack of comprehensive aggregated data of LTC is another obstacle to provide a comprehensive typology work. What is more, the literature mostly covers the cases in Europe or other developed countries without considering the case in developing countries. This study is not ambitious to classify the long-term care in developing countries but inspired by the studies of developed countries. It would classify the community eldercare service with the aim to contribute to the knowledge on community eldercare system design features and also engages with the theoretical discussion of welfare system typology.

When design a typology, a selection of variable is crucial. In the following section, we will discuss the selected variables for typology. Comparative welfare research has made much progress in the measurement of welfare state and change. Even though the discussions about the nature of welfare state and reform direction have not reached consensus, describing welfare state diversity on the low level of abstract by using social rights have been accepted. The use of social rights as systematized concepts allows for the identification of different combinations of two dimensions of social rights, accessibility, and generosity (Kvist, 2007). Dra^7^ c' the won of welfare regime typology (Esping-Andersen, 1990; Clasen and Siegel, 2007; Kraus et al., 2011; Kraus et al., 2010; Ebbinghaus, 2012); we attempt to form a community eldercare service typology based on the system characteristics. We would examine the dimension of accessibility and generosity and attempt to group the cases into different types. The accessibility explains how easy it is to access to publicly finance services. Those variables explain how easy it is to access to publicly financed service (Brodsky et al., 2003; Da Roit et al., 2007). Two features should be considered: (1) Is the community eldercare service is means-tested? The system only targets the poor is means-tested access. In the system that includes the poor and the non-poor; there can be still some degree of means testing if they exclude the population with high income or vary on the level of benefits. (2) Is the community eldercare service is targeting the users with more severe needs or all the residents within the community? At the one extreme, the minimum requirement does not exist, while at the other extreme, they target the ones with need based on the positive evaluation. These two features are examined as the measure of accessibility. As for the dimension of generosity, we will consider the cost-sharing and benefits level (Kraus et al., 2010; Daatland, 2017), (1) does the private household/service users have to share the financial burden for community eldercare service? Private household not only provide inform care but also substantial financial resources for the care provided in institutions and at home. In our typology, the presence or absence of cost sharing as a measure to imply the generosity of the system. Based on the assumption that person in need of care prefer a regime where the private pay is less, we use the this to measure the generosity of the system. If the system is fully funded by the government is the most generous one, and the cost shared among the recipients are in the medium degree of generosity. The service recipients pay all the cost from the pocket is the lest generous one. (2) how much service the system could provide for users? The intensity of care could be seen as a measure of the generosity of community eldercare system. The more intensity of care it provides, the more service capacity is available Whether the community eldercare system provides skill-intensive or low-skill requirement care services. The skill intensive service and more integrated service (e.g., health care and eldercare integrated) the system provide indicate it is more generous. Moreover, how much time and efforts the community eldercare service offer for the recipients. The more time and efforts imply a higher level of generosity.

## The Overview of the Community Eldercare Service Policy Development

This study collects the community eldercare service policy documents from 2000 to 2017 to examine the development trends of community eldercare service proposed by the central government. All the selected sample files are from central government level, including the legislation, regulation, plan, advice, view, and notice. In order to present the trajectory of development, we go through the policy documents in details as follows (see Table 1).

**Table 1:** Policy review of community eldercare service.

**Stage**	**Representative documents**
Traditional family support is the principle for eldercare	Senior citizen rights and interest protection law (1996) The decision on strengthening eldercare (2000)
The initial exploration of community eldercare in policy and practice	The opinion of accelerating the socialization of social welfare (2000) The notice on the pilot campaign of the socialization of eldercare service (2005)
The institutionalization of community eldercare	The opinion on promoting community eldercare service comprehensively (2008) The 12th 5-year plan for the development of the eldercare service (2011)
Pluralist development in community eldercare	The opinion on encouraging private capital into community eldercare service (2012) The view on accelerating the development of eldercare service (2013) The notice of government purchase of eldercare service (2014) The opinion on promoting the integration of healthcare and eldercare (2015) The notice of public finance supporting home-based and community-based eldercare pilot (2016) The 13th 5-year plan for the eldercare service development and system construction

In1996, the Eighth National People's Congress standing committee meeting passed the “senior citizen rights and interest protection law” in the 21st session meeting. This is the only legislation related to the senior citizens, which is also the foundation of the other policies for older people. This legislation emphasizes the family support is the curial part of the eldercare system [National People's Congress (NPC), 1996]. In 2000, the State Council issued “the decision on strengthening eldercare service” and put forward the development goal: the family played a core role in the eldercare; community service padded basic support and private eldercare as the additional part in the whole system. The eldercare system which includes life assistance, health care, fitness, cultural activities, and legal service would be gradually [State Council (SC), 2000]. In 2000, the Ministry of Civil Affairs, State Planning Commission and other 11 departments issued “the opinion on accelerating the socialization of social welfare^1^” and clarified the basic principle of eldercare system: the family was the core and community provided supportive service and the care institution as the additional part. This is China firstly declared to development community service for older adults, children and disabled [Ministry of Civil Affairs (MCA), 2000]. Since then, Shanghai, Beijing, Dalian, Nanjing, Ningbo, and other cities started to explore the community eldercare service practices. In 2005 the Ministry of Civil Affairs issued “the notice on the pilot campaign^2^ of the socialization of eldercare service” and proposed: the eldercare service system should be led by the state and collective investment. The family took the principal responsibility of old people to support. The community service provided basic care for older people and care institution as a supplementary actor to deliver the service [Ministry of Civil Affairs (MCA), 2005]. Since then the community eldercare service as the basic part of the eldercare system had been consolidated in the policy document. All the local government introduced the approach and measures of community eldercare service based on the economic and social condition; some areas even expanded the service to the rural population. In 2008 the National Aging Committee joined with other 10 ministries issued “the opinion on promoting community eldercare service comprehensively,” which was the first specific policy for community eldercare. This policy clarified the principle, basic tasks, and measures and pointed out the direction for the development [National Aging Committee (NAC), 2008]. From 2008 onwards, the development of community eldercare came into the institutionalization and standardization stage. The provinces and cities also initiated the community eldercare service practices; meanwhile, the central government strengthened the guides and regulations for the local pilot practices. In 2011, the State Council issued “The 12th five-year plan for the development of the eldercare service” and proposed to give priority to the community eldercare service development. It put forward the aim of achieving the universal coverage of community eldercare service in urban and 80% coverage rate in county and 50% coverage in rural areas [State Council (SC), 2011]. In 2012, the Ministry of Civil Affairs issued “the opinion on the implement measures of encouraging private capital into community eldercare service” and it proposed that the government would adopt the measures of subsidy, government purchase, coordinated governance, government evaluation to encourage the private capital into the community eldercare development in the urban and rural areas with the aim expand the kinds of services and its accessibility [Ministry of Civil Affairs (MCA), 2012]. In 2013, the State Council issued “the view on accelerating the development of eldercare service” and pointed out that by 2020 China would build up the national eldercare service with family, community, and institution jointly deliver care service for the various demands of older people. The central government would make support policies for private enterprise and social organization to join the care provision and delivery [State Council (SC), 2013]. In 2014, the Ministry of Civil Affairs, Ministry of Finance, the National Development and Reform Commission, and the National Aging Committee joined issued “the notice of government purchase of eldercare service” and requested the local government set up the comprehensive rules to improve the government purchase of eldercare service [Ministry of Finance (MOF), 2014]. It could be found that the community eldercare service shift from a single administrative model to pluralist cooperation model, and the government purchase of eldercare become the important development trend. In 2015, the State Council issued “the opinion on promoting the integration of healthcare and eldercare,” and it explored the integration of health care agency and eldercare agency, encouraging the eldercare service institution to conduct health care service. Also, it supported the social capital to invest in health and eldercare integrated agencies and encouraged the infusion development of healthcare and eldercare [State Council (SC), 2015]. In 2016, the Ministry of Civil Affairs and Ministry of Finance jointly issued “the notice of public finance supporting home-based and community eldercare pilot,” [Ministry of Finance (MOF), 2016] and it aimed to promote the integration of healthcare and eldercare and improve the eldercare service system [Ministry of Civil Affairs (MCA), 2016]. In the same year, the State Council issued “the view on opening up eldercare service market and improving the care quality,” and it put forward the goals of universal coverage of community eldercare service and improving the care quality through the service information platform construction. In 2017, the State Council issued “the 13th 5-year plan for the eldercare service development and system construction,” “and it emphasized the adopting of eldercare service information platform, service call system, emergency medical services to implement the “Internet+^3^” eldercare project [State Council (SC), 2017].

With the issue of intensive government policies, community eldercare service had developed all over the country. According to the social service development bulletin in 2015, the number of community eldercare institution and facility had reached to 26,000, an increase of 16.4% comparing to the previous year [Ministry of Civil Affairs (MCA), 2015]. Apart from the expand and increase of community eldercare, there is some important policy shift. We divide the past two decades into four different stages based on different policy priority. From 1996 to 2000, the eldercare system was mainly based on family support with initial explore of community eldercare. From 2000 to 2005, the traditional idea of family-supporting older people had been challenged, and the consensus that family, community, and society should participate in the eldercare had gradually formed. From 2005 to 2011, the community eldercare was proposed in policy and pilot project was encouraged in practice. The community eldercare underwent a fast development with extensive policy coming out in the central and local level, the role of community eldercare gradually replaced the role of family support and became the principal part of eldercare system. At this stage, extensive regulation regarding the system design feature, standards, evaluation procedures also came out to improve the community eldercare service, which demonstrated the normalization and institutionalization of community eldercare in China. Since 2012, more reform trends were found from the policy documents: the pluralist provisions of eldercare demonstrate the private and social sector were encouraged to join the eldercare service system through diverse mechanisms. Also, the integration of healthcare and eldercare were also promoted by the central government. With the development of big data and technology, advancing the community eldercare service to “smart eldercare^4^” through information platform, technology production was the new trend in the recent. From 2012 to present, this stage was characterized by the pluralist and integrated development with new technology update.

## The Decentralized Practice of Community Eldercare Service in China

After reviewing the policy development in the central level, we will examine the local pilot practices. This study selects four typical practice cases of community eldercare service in different cities, Chongqing, Guangzhou, Qingdao, and Beijing. The four types represent the different local characteristics, ideas, and structure of community eldercare system. Here the four cases will be presented in detail, respectively.

### Chongqing Case

In Chongqing case, the municipal civil affair bureau and a municipal aging committee led the community eldercare service construction, they set up community eldercare service center and collaborated with community committee. The eldercare service center was run in the form of the day care center, community nursing home, and call service center. The community committee was in charge of administrative work transferred from grassroots government and recruited laid-off workers and migrant workers to deliver the eldercare service including domestic help service, meal delivery, daily caring, medical re-habitation, and mental comfort (Qian, 2015). The service users are mostly “three-no^5^” and “empty-nest” older people as well as other old people who are entitled social assistance. Until 2016, a total of 180 million Yuan had been invested in building up 200 urban eldercare service centers, 25 community eldercare information platforms as well as 1,000 rural eldercare nursing (GOV, 2016). The Chongqing practice has built up the community eldercare and guarantee older people are getting aging at home and access to basic aging services from the community with different extents of cost sharing (free, limited pay, or paid service).

### Guangzhou Case

In 2008, Guangzhou and other eastern coastal areas took the lead in the government purchase of community eldercare service. The funding came from public finance to buy the service of social work organization. After several years of efforts, government purchase of community eldercare service has been adopted in other cities; their practice model is mostly based on the Guangzhou practice and in variety. The specific characteristics of government purchase of community eldercare service include: the urban grassroots government is responsible for setting up community eldercare service center; the department of civil affair hold public tenders from social work organization. The successful bidder signs the contracts with government and delivers service to older people in the community. The service programs include domestic service, meal delivery, entrainment activities, daytime nursing, physical health, mental health, information platform, emergency help, etc. (Chen, 2011).

### Qingdao Case

This case is another type of community eldercare service in which the neighbor mutual aid is the main resource for the community eldercare. This model firstly emerged in Qingdao with the aim to assist the empty nester and older people who live alone. Within this model, the community committee organizes the resources of grassroots government, community, and family to support older residents in the community. In particular, the department of civil affair provides facilities and resources for the senior center construction; the community provides the activity space for the activities. Older people who join the mutual-aid team are entitled 100 Yuan subsidize per month from the government. Each mutual-aid team consists of older residents in the community; the younger help, older and the healthier help, the weaker. This mode follows the principle of “voluntary participation, mutual assist, meet up and engage daily” and integrates multiple resources to improve the life quality of older people in the community until 2016 Qingdao has established 1,800 mutual-aid eldercare teams with more than 10,000 participants (Liu, 2017).

### Beijing Case

Beijing as one of the developed cities in China has a more different option in eldercare service development. The retirement community firstly emerged from Beijing. The sun city of Beijing was the first large-scale professional retirement community, which was invented by real estate corporation and the municipal government provided subsidize and tax allowance. The community occupies more than 40 square meters, the interior facilities covering an area of 70,000 square meters. Older people could buy or lease the property in the community and pay the management fees for the access to the high- quality eldercare service. The community provided high quality medical and health service, recreational activities as well as daily domestic help. The resident had to pay the administration fee of 2.5 Yuan per square meter. The rent was 150 Yuan per square meter, and the tenant had to sign the 5-year lease contract with the deposit of 150,000 Yuan (Zhu, 2013).

## The Comparison and Typology of Community Eldercare Service

### The Comparison of the Four Cases

This study is aimed to model the community eldercare service based on the typology method. On the one hand, it would contribute to the knowledge on community eldercare system design; on the other hand, it engages with the theoretical discussion of welfare system typology. We attempt to provide a typology of comprehensive community eldercare service derived from the system features present in different contexts, which are characterized by diverse arrangements of organization, financing and delivering of care. Given the lack of aggregated data of expenditure and provision, this study concentrates on the welfare mix perspective. The concept of the welfare mix has gained particular importance in the field of welfare service. It not only reflects the importance of welfare providers through examining the division of tasks and labor but also account the way how the services are provided, which improve the understanding of welfare service system (Stoy, 2014). This perspective firstly allows for a comprehensive understanding of welfare service beyond policy domain and, second, it takes account the different dimension of welfare service including system characteristics such as principal actors, eligibility criteria, kind of service, financing, and administration mechanism.

Table 2 summaries the distinctive features of each case. The local government assumes primary responsibility for community eldercare under Chongqing and Guangzhou case, a more limited role under the other cases. The formal obligation is assigned to the family in Qingdao and Beijing case, with the government in a subsidiary or residual role. However, as the state is not very generous, the Chongqing case provides the very basic service for an older person, and Guangdong provides service to the people fulfilling the requirements. The low level of care service in Chongqing and the particular principle in Guangdong practice also leaves more responsibility to the family. Also, the family is dominating in eldercare in all cases, perhaps except Beijing case. The family is dominating provider in community eldercare of Qingdao case while the family help is the least intensive if they could afford the eldercare service in Beijing model. It is found that in Chongqing and Guangzhou there is a family-state balance for eldercare, but for the people with extensive needs, the family role will be more prominent in Chongqing case because it only provides very basic care.

**Table 2:** The comparison of community eldercare service models in China.

** 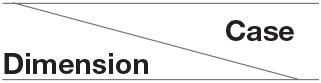 **	**Chongqing**	**Guangzhou**	**Qingdao**	**Beijing**
Principle actor	Local government	Local government	Community elder residents	Enterprise
Eligibility criteria	“Three-no” “Empty nester” Disable old	60+ older people	60+ older people voluntary-based	None
Financing mechanism	Public finance budget	Government special Fund	Government Subsidy	Private investment with limited a tax incentive
Administration mechanism	Public/social programming (government regulation on planning service, personnel, estimated need, population size)	The market mechanism (contracting and competition, competitive tendering)	Volunteerism and mutualism (mutual help between residents)	Market mechanism and consumerism (user fee, maximizing user choice)
Kind of Services	Mainly for free basic service: day care, health care, legal advice and etc.	Cash benefits (service voucher and purchased elder care service	Entertainment and social activities chess, singing, outing, chat and etc.	All aspects of service, Basic necessities of routine life, health care, social care service and other activities.

As for the kind of service, it could be found that the services are the main instruments in all cases and are provided by different actors (local government, the private sector, and non-profit sector). Services in the Chongqing model is predominated publicly provided by grassroots government or residential community. In Guangdong case, the services and cash benefits are jointly provided, and the service is normally outsourced to non-profit organizations and private providers through government purchase service, service voucher, and subsidies. Private-for-profit services are the main instrument in Beijing case and have been encouraged with a tax incentive. The mutual aid services among older residents is a principle in Qingdao case, which supplements the family care.

The access to services as eligibility criteria are different under the four cases. In particular, Chongqing and Qingdao case offer universal access to the services within the residential community while the former has a better capacity to target the people in need while the latter only provide complementary activities for older residents. In Guangdong, the service recipients could use the service after needs assessment. At last, in Beijing case, only the high-income household and person could afford the extensive and expensive eldercare, healthcare, daily care in the residential community, it is very selective based on the income level.

As for the financing model, Chongqing has the public finance budget to the grassroots government and community, which guarantee the care system has more capacity to sustain. While the special government fund takes the second place in the capacity to support the care system, because the special fund is assigned based on government development goal and preference, it could not be as stable as the public budget fund. Qingdao case has limited local government subsidies for mutual help activities; it is unstable and insufficient. Moreover, the Beijing case has a very limited tax incentive for the private providers, which does not support the service users financially.

Finally, as for the administration mechanism, the Chongqing case has a government model in which the local government in charge of planning, financing, managing, delivering and regulation. Moreover, the Guangdong case adopts the governance model in which the local government contract out the service to the non-profit organization and certain for-profit ones and it only has the responsibility of regulation and quality control. Qingdao case is based on the volunteerism, and the service users mutually manage the activities without the involvement of government or other actors except receiving limited subsidize. Beijing mode is the case purely based on market mechanism, and the government assumes the basic regulation responsibility.

### The Typology of the Community Eldercare Service

Based on the analysis framework mentioned above, we classify the four cases into different modes as follows (see Table 3):

1. Lean public service model: in this typology, the government as the dominant actor takes the responsibility of funding, administration, service regulation, and evaluation, also fully involves in the community eldercare system. The service is selective and targets the most vulnerable older people in the community. Also, this type provides the fundamental care for those in need; all the service is a low skill required and time-consuming. However, most of the service is free for the most vulnerable old. Therefore, this model provides minimum basic service for older people in need; we define it as a lean public service mode.
2. Modest universalist model. Guangdong ‘s system provides the service for all the citizen aged 60 and above; it is universal access in nature. However, there is a means-tested for the recipients receiving the service allowance. For most users, they could buy the care service with the regulated price. This mode provides a modest level of care service with some non-profit organizations delivering professional service. Given all the feature above, we conclude this type as a modest universalist mode.
3. Supplementary mutual-aid model. In Qingdao case the mutual-help group and care service is accessible for all the community residents, the financial cost is mutually shared between the participants. While service only plays a supplementary role for older residents, which could not fulfill the absolute needs of older people. Therefore, it is defined as a supplementary mutual-aid model.
4. Comprehensive private model. In Beijing case, the system is run based on private market mechanism, only the high-income household could afford the comprehensive eldercare service in the residential community, but the level of service is the highest compared to other types. We define this type as a comprehensive private model.

**Table 3:** The typology of community eldercare service in China.

**Cases**	**Accessibility**		**Generosity**			**Typology**
	**Means testing**	**Targeting**	**Cost sharing**	**Intensity of care**	**Time and efforts**	
Chongqing	Yes	Selective	No	Low	Low	Residual public service mode
Guangzhou	Yes	Universal	Yes	Medium	Medium	Modest universalist mode
Qingdao	Yes	Universal	Yes	Low	Low	Supplementary mutual-aid mode
Beijing	Yes	Selective	Yes	High	High	Comprehensive private mode

## Conclusion and Discussion

### The Development Trajectory of Community Eldercare Service in China

From the central policy review of community eldercare service, it could be concluded that the development trajectory is from a pilot project on the scratch to the comprehensive community eldercare design. In the past two decades, the central policy initiatives blow out from the 2010s, the number of policy-related with community eldercare service is 76, accounting for 80% of the total policy regulations (2000–2015). Particularly in the consecutive past 3 years, we witnessed the massive release of policy regulation in this issue, the number of policies is 58, accounting for 60% of total regulations. Therefore, it could be argued that the community eldercare service underwent a more structured and comprehensive top-level design in recent years and the central government put more efforts to develop the community eldercare. Also, from the representative policy regulation profile, it could be found that the government involves more in the development of community care' China started from zero community eldercare service while mostly relied on family support for older people and the limited government subsidies for older people' nursing institution in the 1990s. Then it evolved into the second stage of privatization and industrialization of eldercare service in the 2000s. However, coming in the 2010s, the government changed the strategy to construct the community eldercare service system and set up the guidelines for the local pilot practice. However, at this stage, there was no national-level development plan. In the new stage after 2010, the Chinese government had a more organized design for the community eldercare service and encouraged the multi-cooperation (public-private and third sector) to deliver the service. The nationally standardized community eldercare system has not been in shape, but the development direction and pattern have been formed clearly.

### The Policy Implications of Implementing National Community Eldercare Service in China

#### Expand Coverage of Community Eldercare Service

Community eldercare will play a more critical role in the context of shrinking family support and demographic aging. The coverage of community eldercare service has reached 72.5% in urban areas, while rural coverage rate is around 30% (Yan, 2014). Even though the national policy for community eldercare has not come out, the pilot practice has been carried out in most of the provinces, and the acceptance of it is higher than before. Expanding the coverage of community eldercare service is crucial for solving the aging issue in China, particularly the extending it to rural areas is more essential.

#### Multi-Pillar Community Eldercare Service System

After comparing the features of community eldercare service, it is found that the different models have different principle and preference, advantages, and disadvantages. In order to establish a universal national community eldercare service system, it is argued that the multi-pillar system is plausible and catering to the diverse needs of older people. The national community eldercare service design could draw on the different practice models emerging from the local level and build up the multi-pillar community eldercare service system. The zero pillars of could draw on the lean public service model and provide benefits in kinds or voucher for the disadvantaged older people, which is selective coverage with limited government fund. The funding of this pillar from public budget and ensure the minimum care for the vulnerable and disadvantaged old nationwide. The first pillar is basic community eldercare with government planning, funding, and regulation, while non-public sector takes the responsibility service delivery. The eldercare service could be free or with limited pay (depending on the local public finance capacity). The funding of this pillar could come from the health insurance contribution as well as local government budget. The service users are entitled the basic eldercare service provided at the community, such as senior activity center facilities, life assistance programs, health habitation service and so on. These services could be paid by government purchase and the personal health insurance contribution. The second pillar is the private pillar with government regulation and tax allowance or incentives, and the private sector takes the responsibility of funding and service delivery. The customers could use the service by private pay. Given the urban-rural development gap, I could argue that the rural areas at least should be covered by the zero-floor pillar and provide basic support for the most disadvantaged older people.

#### Coordinated Policy Suggestions for Multi-Pillar Model Development

First, the balance between institutional eldercare and community eldercare. In the first pillar, more institutional care agencies should be introduced into the community; they could provide professional care, medical care, and other activities. It could improve the service quality and sustainable development of community eldercare. The institutional care agencies as the service delivery partners for government provides the primary daycare and healthcare for the community residents.

Second, the balance between the public sector and the private sector. As mentioned before, in the multi-pillar system, the government should encourage and promote the private capital into commercial eldercare service in the second pillar. For instance, public nursing could be privatized and transfer to private eldercare institution. More tax incentives and allowance for the age-friendly property development.

Finally, the balance between formal and informal caregiver. In order to increase the care service level, professional caregivers and nurses are needed for the community eldercare service. Coming with the demographic aging, the silver economy is the shortage of professional staff providing eldercare. Physicians, nurses, social worker, physical therapists, nutritionists as the core service staff of eldercare system are the foundations of building comprehensive community eldercare. The talent policy and incentives for these groups should be implemented to attract them coming to this service industry. Also, integrating the health professional staff into the community care service system on a part-time basis, they could join the social organization, eldercare society or voluntary organization to deliver better service for the community. Also, developing the formal community eldercare service should also take informal care into consideration. In the Chinese welfare regime, the family support is crucial in the eldercare, how to integrate the family care into the community eldercare system and support the informal care from family is another important direction. The subsidy for family caregivers or means-tested care service provision for the disadvantaged household is an option.

## Author Contributions

LZ contributed to the design of the research and the analysis of the results and the writing of the manuscript. JY contributed to the data collection from the policy review and part of data analysis.

### Conflict of Interest Statement

The authors declare that the research was conducted in the absence of any commercial or financial relationships that could be construed as a potential conflict of interest.

## Footnotes

^1^Socialization of social welfare means that the welfare system is not only the responsibility of state as in planned economy, multiple actors including social sector, private sector join the welfare service provision and delivery.

^2^Pilot campaign in China context is a special policy process. Given the fact that China is undergoing transition from plan economy to market economy, any reform or innovation started from new. The pilot campaign allows the local governments carry out the pilot practice based on local conditions under the central government's guideline. After several years of pilot, the central government would initiate national program based on local practices and experiences.

^3^Adopting internet technology to develop APP for older adults and facilitate them to make doctor appointment, food delivery, home service, etc. by internet technique.

^4^Smart eldercare refers to the use of information technology (IT) to develop information platform for older adults, communities, and institutions to provide effective and efficient eldercare services. The comprehensive service platforms of “eldercare” and “health” connecting service providers, individuals, and families to meet the diverse and multi-level needs of older adults. It is an important reform direction in eldercare.

^5^“Three noes” refers to people who have no ability to work, no income, and no means to support dependents.
